# Cs_3_Sm_7_Se_12_

**DOI:** 10.1107/S1600536811051919

**Published:** 2011-12-07

**Authors:** Christof Schneck, Andreas Elbe, Christian M. Schurz, Thomas Schleid

**Affiliations:** a Institut für Anorganische Chemie, Universität Stuttgart, Pfaffenwaldring 55, 70569 Stuttgart, Germany

## Abstract

The title compound, tricaesium hepta­samarium(III) dodeca­selenide, is setting a new starting point for realization of the channel structure of the Cs_3_ *M*_7_Se_12_ series, now with *M* = Sm, Gd–Er. This Cs_3_Y_7_Se_12_-type arrangement is structurally based on the *Z*-type sesquiselenides *M*_2_Se_3_ adopting the Sc_2_S_3_ structure. Thus, the structural set-up of Cs_3_Sm_7_Se_12_ consists of edge- and vertex-connected [SmSe_6_]^9−^ octa­hedra [*d*_Ø_(Sm^3+^ – Se^2−^) = 2.931 Å], forming a rock-salt-related network [Sm_7_Se_12_]^3−^ with channels along [001] that are apt to take up monovalent cations (here Cs^+^) with coordination numbers of 7 + 1 for one and of 6 for the second cation. The latter cation has a trigonal–prismatic coordination and shows half-occupancy, resulting in an impossible short distance [2.394 (4) Å] between symmetrically coupled Cs^+^ cations of the same kind. While one Sm atom occupies *Wyckoff* position 2*b* with site symmetry ..2/*m*, all other 11 crystallographically different atoms (namely 2 × Cs, 3 × Sm and 6 × Se) are located at *Wyckoff* positions 4*g* with site symmetry ..*m*.

## Related literature

For prototypic Cs_3_Y_7_Se_12_ or Rb_3_Yb_7_Se_12_, see: Folchnandt & Schleid (1996 ▶); Kim *et al.* (1996 ▶). For other representatives of the *A*_3_ *M*_7_ *Ch*_12_ series, see: Folchnandt & Schleid (1997 ▶, 1998 ▶, 2000 ▶); Tougaît *et al.* (2001 ▶); Lissner *et al.* (2002 ▶). A detailed description of the relation between the crystal structures of the Cs_3_ *M*_7_Se_12_ series and Z-type Sc_2_ *Ch*_3_ (Dismukes & White, 1964 ▶) is provided by Folchnandt & Schleid (1998 ▶).

## Experimental

### 

#### Crystal data

Cs_3_Sm_7_Se_12_
*M* *_r_* = 2398.70
Orthorhombic, 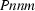

*a* = 13.0387 (9) Å
*b* = 26.6742 (19) Å
*c* = 4.2351 (3) Å
*V* = 1472.95 (18) Å^3^
*Z* = 2
Mo *K*α radiation
μ = 32.19 mm^−1^
*T* = 293 K
0.10 × 0.07 × 0.05 mm

#### Data collection

Stoe IPDS-I diffractometer
Absorption correction: numerical (*X-SHAPE*; Stoe & Cie, 1999 ▶) *T*_min_ = 0.115, *T*_max_ = 0.216
15080 measured reflections
2135 independent reflections
1587 reflections with *I* > 2σ(*I*)
*R*_int_ = 0.065

#### Refinement

*R*[*F* ^2^ > 2σ(*F* ^2^)] = 0.035
*wR*(*F* ^2^) = 0.072
*S* = 0.97
2135 reflections
72 parameters
Δρ_max_ = 2.09 e Å^−3^
Δρ_min_ = −1.78 e Å^−3^

### 

Data collection: *DIF4* (Stoe & Cie, 1992 ▶); cell refinement: *DIF4*; data reduction: *REDU4* (Stoe & Cie, 1992 ▶); program(s) used to solve structure: *SHELXS97* (Sheldrick, 2008 ▶); program(s) used to refine structure: *SHELXL97* (Sheldrick, 2008 ▶); molecular graphics: *DIAMOND* (Brandenburg, 2006 ▶); software used to prepare material for publication: *SHELXL97*.

## Supplementary Material

Crystal structure: contains datablock(s) I, global. DOI: 10.1107/S1600536811051919/wm2561sup1.cif

Structure factors: contains datablock(s) I. DOI: 10.1107/S1600536811051919/wm2561Isup2.hkl

Additional supplementary materials:  crystallographic information; 3D view; checkCIF report

## Figures and Tables

**Table 1:** Selected bond lengths (Å)

Cs1—Se4^i^	3.6071 (12)
Cs1—Se4^ii^	3.6071 (12)
Cs1—Se6^ii^	3.7129 (12)
Cs1—Se6^i^	3.7129 (12)
Cs1—Se3^iii^	3.7639 (14)
Cs1—Se5^i^	3.8053 (12)
Cs1—Se5^ii^	3.8053 (12)
Cs1—Se1	4.5421 (14)
Cs2—Se2^ii^	3.5286 (16)
Cs2—Se2^i^	3.5286 (16)
Cs2—Se2^v^	3.6917 (17)
Cs2—Se2^vi^	3.6917 (17)
Cs2—Se5^iii^	3.719 (2)
Cs2—Se6^iii^	3.924 (2)

Symmetry codes: (i) 
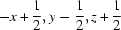
; (ii) 
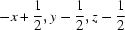
; (iii) 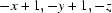; (iv) 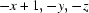; (v) 
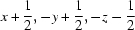
; (vi) 
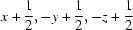
.

**Fig. 1.**
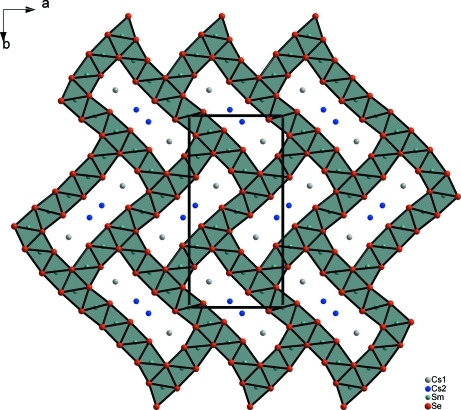
Channel-structure representation of Cs3Sm7Se12 as octahedral framework with indicated unit cell.

**Fig. 2.**
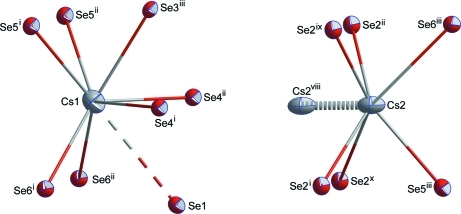
Coordination spheres of the Cs1+ (left) and Cs2+ (right) cations in Cs3Sm7Se12. Displacement ellipsoids are drawn at the 90% probability level. Symmetry codes: (i) -x+1/2, y-1/2, z+1/2; (ii) -x+1/2, y-1/2, z-1/2; (iii) -x+1, -y+1, -z; (viii) -x+1, -y, -z; (ix) x+1/2, -y+1/2, -z-1/2; (x) x+1/2, -y+1/2, -z+1/2.

**Fig. 3.**
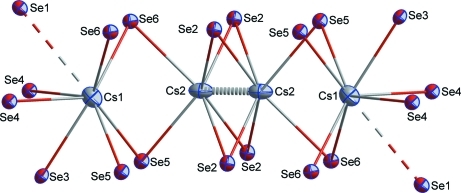
Interplay of the Cs+ cations situated in the triple-channels of the crystal structure of Cs3Sm7Se12. Displacement ellipsoids are drawn at the 90% probability level.

## supplementary crystallographic information

### Comment

Cs_3_Sm_7_Se_12_ crystallizes isotypically to the large family of ternary *A*_3_*M*_7_*Ch*_12_ representatives with a channel-like structure. For *Ch* = S, *A* = K, Rb, *M* = Er, see: Lissner *et al.* (2002); for *Ch* = Se, *A* = Rb, *M* = Dy, Yb, see: Folchnandt & Schleid (2000), Kim *et al.* (1996); for *Ch* = Se, *A* = Cs, *M* = Y, Gd – Er, see: Folchnandt & Schleid (1996, 1997, 1998); for *Ch* = Te, *A* = Cs, *M* = Sm, Gd, Tb, see: Tougaît *et al.* (2001).

In the title compound, [SmSe_6_]^9-^ octahedra (*d*(Sm^3+^–Se^2-^) = 2.8578 (9)–3.0614 (13) Å) are connected via edges and corners to form a [Sm_7_Se_12_]^3-^ network with triple-channels occupied by Cs^+^ cations (Fig. 1). This network represents a defect rock-salt-type structure strongly related to that of the *Z*-type sesquiselenides *M*_2_Se_3_ (Dismukes & White, 1964) according to the formula [□]_4_[*M*]_8_[Se]_12_. In tricaesium heptasamarium(III) dodecaselenide three Cs^+^ cations replace one Sm^3+^ for charge balance. The triple-channels are arranged in a herringbone pattern and run through the structure parallel to [001]. They are filled with two crystallographically different Cs^+^ cations (Fig. 2). While Cs1^+^ exhibits a coordination number of 7+*1* with an extra secondary contact (*d*(Cs1^+^–Se^2-^) = 3.6071 (12)–3.8053 (12) Å and 4.5421 (14) Å; Fig. 2, *left*), the Cs2^+^ cations have only six selenide anions as nearest neighbours in the shape of a trigonal prism (*d*(Cs2^+^–Se^2-^) = 3.5286 (16) – 3.924 (2) Å; Fig. 2, *right*). Owing to the very close distances between these Cs2^+^ cations (*d*(Cs2^+^···Cs2^+^) = 2.394 (4) Å) only a half-occupation of this position is possible (Fig. 2, *right* and Fig. 3) and stoichiometrically meaningful.

### Experimental

Yellow, transparent, needle-shaped single crystals of Cs_3_Sm_7_Se_12_ were obtained as the main product of a reaction between 0.10 g Sm, 0.08 g Se and 0.50 g CsCl added as flux and caesium source upon heating at 1073 K for 10 days in a sealed, evacuated fused-silica vessel.

### Refinement

In the final difference Fourier map the highest peak is 1.24 Å away from Se2 and the deepest hole is located 0.83 Å away from Sm2.

### Crystal data

**Table:**

Cs_3_Sm_7_Se_12_	*F*(000) = 2014
*M**_r_* = 2398.70	*D*_x_ = 5.408 Mg m^−^^3^
Orthorhombic, *P**n**n**m*	Mo *K*α radiation, λ = 0.71069 Å
Hall symbol: -P 2 2n	Cell parameters from 5000 reflections
*a* = 13.0387 (9) Å	θ = 2.1–29.3°
*b* = 26.6742 (19) Å	µ = 32.19 mm^−^^1^
*c* = 4.2351 (3) Å	*T* = 293 K
*V* = 1472.95 (18) Å^3^	Needle, yellow
*Z* = 2	0.10 × 0.07 × 0.05 mm

### Data collection

**Table:**

Stoe IPDS-I diffractometer	2135 independent reflections
Radiation source: fine-focus sealed tube	1587 reflections with *I* > 2σ(*I*)
graphite	*R*_int_ = 0.065
imaging plate detector system scans	θ_max_ = 29.0°, θ_min_ = 2.8°
Absorption correction: numerical (*X-SHAPE*; Stoe & Cie, 1999)	*h* = −17→17
*T*_min_ = 0.115, *T*_max_ = 0.216	*k* = −36→36
15080 measured reflections	*l* = −5→5

### Refinement

**Table:**

Refinement on *F*^2^	Primary atom site location: structure-invariant direct methods
Least-squares matrix: full	Secondary atom site location: difference Fourier map
*R*[*F*^2^ > 2σ(*F*^2^)] = 0.035	*w* = 1/[σ^2^(*F*_o_^2^) + (0.0371*P*)^2^] where *P* = (*F*_o_^2^ + 2*F*_c_^2^)/3
*wR*(*F*^2^) = 0.072	(Δ/σ)_max_ = 0.014
*S* = 0.97	Δρ_max_ = 2.09 e Å^−^^3^
2135 reflections	Δρ_min_ = −1.78 e Å^−^^3^
72 parameters	Extinction correction: *SHELXL97* (Sheldrick, 2008), Fc^*^=kFc[1+0.001xFc^2^λ^3^/sin(2θ)]^-1/4^
0 restraints	Extinction coefficient: 0.00017 (4)

### Special details

**Table:**

Geometry. All esds (except the esd in the dihedral angle between two l.s. planes) are estimated using the full covariance matrix. The cell esds are taken into account individually in the estimation of esds in distances, angles and torsion angles; correlations between esds in cell parameters are only used when they are defined by crystal symmetry. An approximate (isotropic) treatment of cell esds is used for estimating esds involving l.s. planes.
Refinement. Refinement of F^2^ against ALL reflections. The weighted R-factor wR and goodness of fit S are based on F^2^, conventional R-factors R are based on F, with F set to zero for negative F^2^. The threshold expression of F^2^ > 2sigma(F^2^) is used only for calculating R-factors(gt) etc. and is not relevant to the choice of reflections for refinement. R-factors based on F^2^ are statistically about twice as large as those based on F, and R- factors based on ALL data will be even larger.

### Fractional atomic coordinates and isotropic or equivalent isotropic displacement parameters (Å^2^)

**Table:**

	*x*	*y*	*z*	*U*_iso_*/*U*_eq_	Occ. (<1)
Cs1	0.28796 (6)	0.36600 (3)	0.0000	0.0252 (2)	
Cs2	0.56532 (14)	0.03154 (7)	0.0000	0.0311 (5)	0.50
Sm1	0.0000	0.0000	0.5000	0.0124 (2)	
Sm2	0.21848 (5)	0.08314 (2)	0.0000	0.01316 (15)	
Sm3	0.40590 (4)	0.71214 (2)	0.0000	0.01224 (15)	
Sm4	0.07792 (5)	0.68240 (2)	0.0000	0.01271 (15)	
Se1	0.25592 (8)	0.19644 (4)	0.0000	0.0125 (3)	
Se2	0.12980 (9)	0.57736 (4)	0.0000	0.0142 (3)	
Se3	0.43019 (9)	0.60350 (4)	0.0000	0.0129 (3)	
Se4	0.05307 (8)	0.78890 (4)	0.0000	0.0133 (3)	
Se5	0.15051 (9)	0.98065 (4)	0.0000	0.0142 (3)	
Se6	0.42742 (9)	0.82140 (4)	0.0000	0.0133 (3)	

### Atomic displacement parameters (Å^2^)

**Table:**

	*U*^11^	*U*^22^	*U*^33^	*U*^12^	*U*^13^	*U*^23^
Cs1	0.0214 (4)	0.0295 (4)	0.0247 (6)	−0.0002 (3)	0.000	0.000
Cs2	0.0266 (9)	0.0273 (9)	0.0393 (14)	0.0114 (7)	0.000	0.000
Sm1	0.0147 (4)	0.0109 (4)	0.0116 (6)	−0.0030 (3)	0.000	0.000
Sm2	0.0153 (3)	0.0135 (3)	0.0107 (4)	−0.0027 (2)	0.000	0.000
Sm3	0.0137 (3)	0.0117 (3)	0.0113 (4)	−0.0018 (2)	0.000	0.000
Sm4	0.0131 (3)	0.0130 (3)	0.0121 (4)	0.0034 (2)	0.000	0.000
Se1	0.0123 (5)	0.0153 (5)	0.0101 (7)	−0.0004 (4)	0.000	0.000
Se2	0.0147 (6)	0.0142 (5)	0.0137 (8)	0.0007 (4)	0.000	0.000
Se3	0.0154 (5)	0.0096 (5)	0.0138 (7)	0.0007 (4)	0.000	0.000
Se4	0.0144 (5)	0.0128 (5)	0.0126 (8)	0.0000 (4)	0.000	0.000
Se5	0.0155 (5)	0.0129 (5)	0.0141 (8)	−0.0004 (4)	0.000	0.000
Se6	0.0140 (5)	0.0134 (5)	0.0124 (8)	0.0008 (4)	0.000	0.000

### Geometric parameters (Å, °)

**Table:**

Cs1—Se4^i^	3.6071 (12)	Sm1—Se5^xi^	2.9328 (8)
Cs1—Se4^ii^	3.6071 (12)	Sm1—Se5^xii^	2.9328 (8)
Cs1—Se6^ii^	3.7129 (12)	Sm1—Se5^xiii^	2.9328 (8)
Cs1—Se6^i^	3.7129 (12)	Sm2—Se5^xiii^	2.8738 (13)
Cs1—Se3^iii^	3.7639 (14)	Sm2—Se2^i^	2.9020 (10)
Cs1—Se5^i^	3.8053 (12)	Sm2—Se2^ii^	2.9020 (10)
Cs1—Se5^ii^	3.8053 (12)	Sm2—Se3^ii^	2.9217 (9)
Cs1—Se1	4.5421 (14)	Sm2—Se3^i^	2.9217 (9)
Cs1—Cs1^iv^	4.2351 (3)	Sm2—Se1	3.0614 (13)
Cs1—Cs1^v^	4.2351 (3)	Sm3—Se4^xiv^	2.8578 (9)
Cs2—Cs2^vi^	2.394 (4)	Sm3—Se4^xv^	2.8578 (9)
Cs2—Se2^ii^	3.5286 (16)	Sm3—Se3	2.9152 (13)
Cs2—Se2^i^	3.5286 (16)	Sm3—Se6	2.9279 (13)
Cs2—Se2^vii^	3.6917 (17)	Sm3—Se1^xvi^	3.0185 (9)
Cs2—Se2^viii^	3.6917 (17)	Sm3—Se1^xvii^	3.0185 (9)
Cs2—Se5^iii^	3.719 (2)	Sm4—Se4	2.8591 (13)
Cs2—Se6^iii^	3.924 (2)	Sm4—Se2	2.8823 (13)
Cs2—Sm2^vi^	4.1597 (18)	Sm4—Se6^xviii^	2.8888 (9)
Sm1—Se3^i^	2.9070 (11)	Sm4—Se6^xix^	2.8888 (9)
Sm1—Se3^ix^	2.9070 (11)	Sm4—Se1^xvii^	3.0526 (9)
Sm1—Se5^x^	2.9328 (8)	Sm4—Se1^xvi^	3.0526 (9)
			
Se4^i^—Cs1—Se4^ii^	71.90 (3)	Se2^ii^—Sm2—Se1	86.77 (3)
Se4^i^—Cs1—Se6^ii^	125.93 (3)	Se3^ii^—Sm2—Se1	85.54 (3)
Se4^ii^—Cs1—Se6^ii^	85.24 (2)	Se3^i^—Sm2—Se1	85.54 (3)
Se4^i^—Cs1—Se6^i^	85.24 (2)	Se4^xiv^—Sm3—Se4^xv^	95.63 (4)
Se4^ii^—Cs1—Se6^i^	125.93 (3)	Se4^xiv^—Sm3—Se3	85.26 (3)
Se6^ii^—Cs1—Se6^i^	69.55 (3)	Se4^xv^—Sm3—Se3	85.26 (3)
Se4^i^—Cs1—Se3^iii^	64.04 (3)	Se4^xiv^—Sm3—Se6	86.87 (3)
Se4^ii^—Cs1—Se3^iii^	64.04 (3)	Se4^xv^—Sm3—Se6	86.87 (3)
Se6^ii^—Cs1—Se3^iii^	143.874 (16)	Se3—Sm3—Se6	168.27 (4)
Se6^i^—Cs1—Se3^iii^	143.874 (16)	Se4^xiv^—Sm3—Se1^xvi^	171.05 (4)
Se4^i^—Cs1—Se5^i^	90.60 (2)	Se4^xv^—Sm3—Se1^xvi^	87.03 (2)
Se4^ii^—Cs1—Se5^i^	131.59 (3)	Se3—Sm3—Se1^xvi^	86.44 (3)
Se6^ii^—Cs1—Se5^i^	137.25 (3)	Se6—Sm3—Se1^xvi^	101.84 (3)
Se6^i^—Cs1—Se5^i^	95.72 (2)	Se4^xiv^—Sm3—Se1^xvii^	87.03 (2)
Se3^iii^—Cs1—Se5^i^	67.69 (3)	Se4^xv^—Sm3—Se1^xvii^	171.05 (4)
Se4^i^—Cs1—Se5^ii^	131.59 (3)	Se3—Sm3—Se1^xvii^	86.44 (3)
Se4^ii^—Cs1—Se5^ii^	90.60 (2)	Se6—Sm3—Se1^xvii^	101.84 (3)
Se6^ii^—Cs1—Se5^ii^	95.72 (2)	Se1^xvi^—Sm3—Se1^xvii^	89.10 (3)
Se6^i^—Cs1—Se5^ii^	137.25 (3)	Se4—Sm4—Se2	172.93 (4)
Se3^iii^—Cs1—Se5^ii^	67.69 (3)	Se4—Sm4—Se6^xviii^	87.59 (3)
Se5^i^—Cs1—Se5^ii^	67.63 (2)	Se2—Sm4—Se6^xviii^	97.20 (3)
Se2^ii^—Cs2—Se2^vii^	95.31 (3)	Se4—Sm4—Se6^xix^	87.59 (3)
Se2^i^—Cs2—Se2^vii^	141.35 (6)	Se2—Sm4—Se6^xix^	97.20 (3)
Se2^ii^—Cs2—Se2^viii^	141.35 (6)	Se6^xviii^—Sm4—Se6^xix^	94.28 (4)
Se2^i^—Cs2—Se2^viii^	95.31 (3)	Se4—Sm4—Se1^xvii^	87.63 (3)
Se2^vii^—Cs2—Se2^viii^	70.00 (4)	Se2—Sm4—Se1^xvii^	87.29 (3)
Se2^ii^—Cs2—Se5^iii^	138.46 (3)	Se6^xviii^—Sm4—Se1^xvii^	174.23 (4)
Se2^i^—Cs2—Se5^iii^	138.46 (3)	Se6^xix^—Sm4—Se1^xvii^	88.74 (2)
Se2^vii^—Cs2—Se5^iii^	72.81 (4)	Se4—Sm4—Se1^xvi^	87.63 (3)
Se2^viii^—Cs2—Se5^iii^	72.81 (4)	Se2—Sm4—Se1^xvi^	87.29 (3)
Se2^ii^—Cs2—Se6^iii^	70.80 (4)	Se6^xviii^—Sm4—Se1^xvi^	88.74 (2)
Se2^i^—Cs2—Se6^iii^	70.80 (4)	Se6^xix^—Sm4—Se1^xvi^	174.23 (4)
Se2^vii^—Cs2—Se6^iii^	141.36 (3)	Se1^xvii^—Sm4—Se1^xvi^	87.85 (3)
Se2^viii^—Cs2—Se6^iii^	141.36 (3)	Sm3^ii^—Se1—Sm3^i^	89.10 (3)
Se5^iii^—Cs2—Se6^iii^	93.63 (5)	Sm3^ii^—Se1—Sm4^i^	178.80 (4)
Se3^i^—Sm1—Se3^ix^	180.000 (14)	Sm3^i^—Se1—Sm4^i^	91.519 (12)
Se3^i^—Sm1—Se5^x^	92.43 (3)	Sm3^ii^—Se1—Sm4^ii^	91.519 (12)
Se3^ix^—Sm1—Se5^x^	87.57 (3)	Sm3^i^—Se1—Sm4^ii^	178.80 (4)
Se3^i^—Sm1—Se5^xi^	87.57 (3)	Sm4^i^—Se1—Sm4^ii^	87.85 (3)
Se3^ix^—Sm1—Se5^xi^	92.43 (3)	Sm3^ii^—Se1—Sm2	91.46 (3)
Se5^x^—Sm1—Se5^xi^	180.00 (4)	Sm3^i^—Se1—Sm2	91.46 (3)
Se3^i^—Sm1—Se5^xii^	92.43 (3)	Sm4^i^—Se1—Sm2	89.55 (3)
Se3^ix^—Sm1—Se5^xii^	87.57 (3)	Sm4^ii^—Se1—Sm2	89.55 (3)
Se5^x^—Sm1—Se5^xii^	92.44 (3)	Sm4—Se2—Sm2^xvii^	96.22 (3)
Se5^xi^—Sm1—Se5^xii^	87.56 (3)	Sm4—Se2—Sm2^xvi^	96.22 (3)
Se3^i^—Sm1—Se5^xiii^	87.57 (3)	Sm2^xvii^—Se2—Sm2^xvi^	93.72 (4)
Se3^ix^—Sm1—Se5^xiii^	92.43 (3)	Sm1^viii^—Se3—Sm3	167.99 (5)
Se5^x^—Sm1—Se5^xiii^	87.56 (3)	Sm1^viii^—Se3—Sm2^xvi^	91.79 (3)
Se5^xi^—Sm1—Se5^xiii^	92.44 (3)	Sm3—Se3—Sm2^xvi^	96.47 (3)
Se5^xii^—Sm1—Se5^xiii^	180.0	Sm1^viii^—Se3—Sm2^xvii^	91.79 (3)
Se5^xiii^—Sm2—Se2^i^	99.19 (3)	Sm3—Se3—Sm2^xvii^	96.47 (3)
Se5^xiii^—Sm2—Se2^ii^	99.19 (3)	Sm2^xvi^—Se3—Sm2^xvii^	92.90 (4)
Se2^i^—Sm2—Se2^ii^	93.72 (4)	Sm3^xviii^—Se4—Sm3^xix^	95.63 (4)
Se5^xiii^—Sm2—Se3^ii^	88.41 (3)	Sm3^xviii^—Se4—Sm4	93.81 (3)
Se2^i^—Sm2—Se3^ii^	172.31 (4)	Sm3^xix^—Se4—Sm4	93.81 (3)
Se2^ii^—Sm2—Se3^ii^	86.18 (2)	Sm2^xx^—Se5—Sm1^xxi^	92.23 (3)
Se5^xiii^—Sm2—Se3^i^	88.41 (3)	Sm2^xx^—Se5—Sm1^xx^	92.23 (3)
Se2^i^—Sm2—Se3^i^	86.18 (2)	Sm1^xxi^—Se5—Sm1^xx^	92.44 (3)
Se2^ii^—Sm2—Se3^i^	172.31 (4)	Sm4^xiv^—Se6—Sm4^xv^	94.28 (4)
Se3^ii^—Sm2—Se3^i^	92.90 (4)	Sm4^xiv^—Se6—Sm3	91.72 (3)
Se5^xiii^—Sm2—Se1	171.21 (4)	Sm4^xv^—Se6—Sm3	91.72 (3)
Se2^i^—Sm2—Se1	86.77 (3)		

Symmetry codes: (i) −*x*+1/2, *y*−1/2, *z*+1/2; (ii) −*x*+1/2, *y*−1/2, *z*−1/2; (iii) −*x*+1, −*y*+1, −*z*;  (iv) *x*, *y*, *z*−1; (v) *x*, *y*, *z*+1; (vi) −*x*+1, −*y*, −*z*; (vii) *x*+1/2, −*y*+1/2, −*z*−1/2; (viii) *x*+1/2, −*y*+1/2, −*z*+1/2; (ix) *x*−1/2, −*y*+1/2, −*z*+1/2; (x) −*x*, −*y*+1, −*z*; (xi) *x*, *y*−1, *z*+1; (xii) −*x*, −*y*+1, −*z*+1; (xiii) *x*, *y*−1, *z*; (xiv) *x*+1/2, −*y*+3/2, −*z*+1/2; (xv) *x*+1/2, −*y*+3/2, −*z*−1/2; (xvi) −*x*+1/2, *y*+1/2, *z*−1/2; (xvii) −*x*+1/2, *y*+1/2, *z*+1/2; (xviii) *x*−1/2, −*y*+3/2, −*z*−1/2; (xix) *x*−1/2, −*y*+3/2, −*z*+1/2; (xx) *x*, *y*+1, *z*; (xxi) *x*, *y*+1, *z*−1.
